# Role of chemotherapy in the management of primary rectal lymphoma: a case report and review of the literature

**DOI:** 10.1186/1757-1626-2-9373

**Published:** 2009-12-22

**Authors:** Nabil Ismaili, Youssef Bensouda, Nawfel Mellas, Hassan Errihani

**Affiliations:** 1 Department of Medical Oncology, National Institute of Oncology, Rabat, Morocco

## Abstract

**Introduction:**

Primary rectal lymphoma is a rare disease. In this paper we present an unusual case of primary rectal lymphoma which was managed with chemotherapy and discussed by a thorough review of the related literature.

**Cases presentation:**

An 85-years-male patient was diagnosed in Sidi Mohammed Ben Abdellah Hospital as having diffuse large B-cell lymphoma of the rectum at a bulky stage two. This patient was managed successfully with 8 treatment cycles of Cyclophosphamide 750 mg/m2 at day 1 of each cycle, Doxorubicin (50 mg/m2 in the first 4 cycles and 25 mg/m2 in the subsequent cycles) at day 1 of each cycle, Vincristine 1.4 mg/m^2 ^at day 1 of each cycle, and prednisone 50 mg/m^2 ^at day 1 to 5 of each cycle.

**Conclusion:**

The optimal treatment of primary rectal lymphoma needs more research studying to be achieved.

## Introduction

Lymphomas are malignant neoplasms of the lymphocyte cell lines. They mainly involve lymph nodes, spleen and other non-haemopoietic tissues. They are mainly classified as either Hodgkin's or non-Hodgkin's lymphomas (NHL), and of either B-lymphocyte or T-lymphocyte origin. According to estimates, in 2008, 66120 new cases of NHL occurred in United State. Gastrointestinal NHL account for 4% to 20% of all NHL. Approximately 0.2 to 0.6% of malignant lymphomas arise in the colorectal tract [1]. Primary rectal NHL is rare accounting for 0.2% of all rectal malignancies [2]. The diffuse large B-cell lymphoma (DLBCL) is the most common type of primary colorectal non Hodgkin lymphoma (NHL) [1]. In this paper, we will present a case of primary rectal diffuse large B-cell lymphoma at bulky stage II in an 85 year old man with unusual presentation and focus on the role of chemotherapy according to the English literature.

## Case presentation

**Figure 1:**
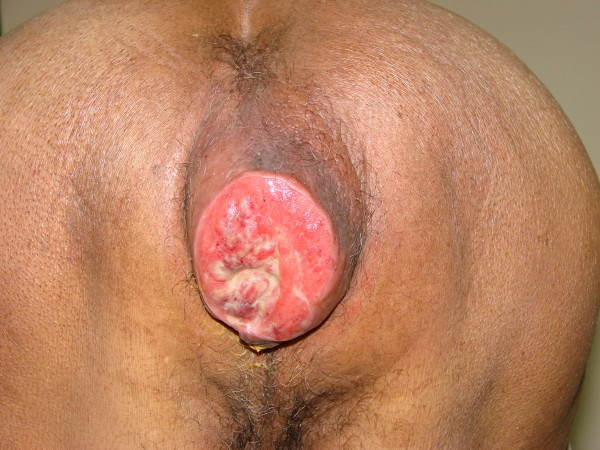
**Peri-anal ulcerative-vegetative tumour measured 10 × 10 × 4 cm**.

**Figure 2:**
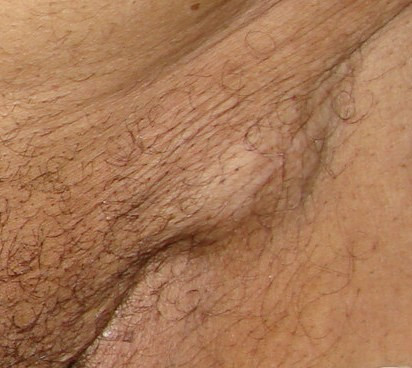
**Enlarged lymph nodes in the inguinal area (see arrow)**.

**Figure 3:**
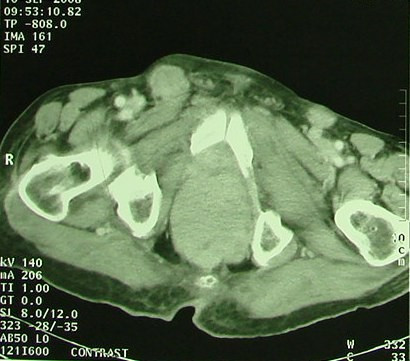
**Pelvic computed tomography scan performed before chemotherapy treatment shows the Bulky anorectal process (see arrows)**.

**Figure 4:**
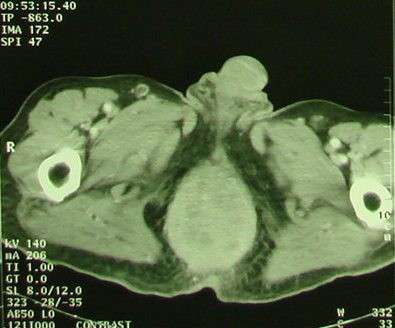
**Pelvic computed tomography scan performed before chemotherapy treatment shows the Bulky anorectal process with enlarged lymph nodes in the inguinal areas (see arrows)**.

**Figure 5:**
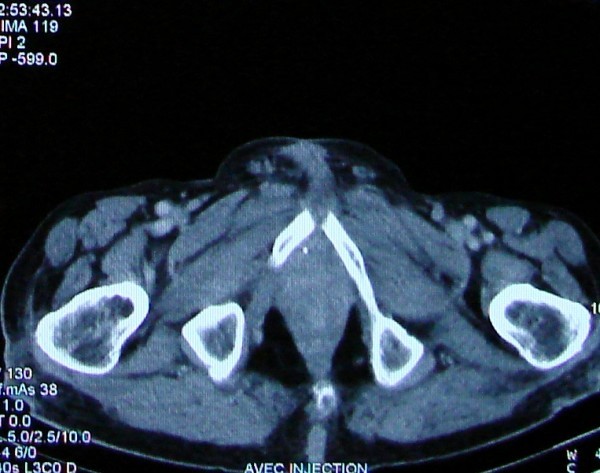
**Pelvic computed tomography scan performed after the end of chemotherapy shows complete radiological response of the ano-rectal tumour**.

A 85-years-old Morrocan Arab man was admitted to the National Institute of Oncology Hospital with alteration of health (asthenia without weight loss), systemic symptoms (Fever, shudder, profuse sweat), yellow rectal secretions for 18 months, and enlarged tumour in the peri-anal region and rectal tenesmus for 3 months. The tumour mass increased rapidly in volume and was associated with ulceration at the surface. The patient suffered equally of urinary signs of dysuria and pollakiuria. The evolution was marked by the apparition of inguinal lymph nods 3 months ago. His past medical history did not include co-morbidities, polypharmacy, malnutrition or dependency. At presentation, the patient had an ECOG performance status equal to 2.0. Physical examination of the pelvis showed a heavily peri-anal ulcerative-vegetative mass measuring 10 × 10 × 4 cm (Figure 1). The examination of ganglionic aereas showed bilateral lymph nodes in the inguinal region measuring 2 × 2 cm (Figure 2). Pelvic computed tomography scan showed a circumferential anorectal tissular process associated with retro rectal and bilateral inguinal lymph nodes. The tumour infiltrated the mesorectal fate, provoked a thickness of the fascias and came in contact with obturator muscles (Figure 3 and 4). The rectal biopsy was performed. Histological and immunohistochemistry studies showed DLBCL of the rectum according to the Revised European-American Classification of Lymphoid Neoplasms/World Health Organisation classification of lymphoid neoplasms (REAL/WHO). Most of the neoplasic cells were positive for CD-20 and for leukocyte common antigen (LCA) antibody. Computed tomography of the chest and abdomen was normal. The erythrocyte sedimentation rate was increased to 70 mm in the first hour. The white blood cell counts were within normal limits. A bone marrow biopsy showed no abnormalities. The patient was staged IIEXBb according to the Ann Arbor Staging system. Before first chemotherapy cycle, the cardiac fraction ejection was normal and was equal to 75%. The patient received 8 cycles of Cyclophosphamide 750 mg/m^2 ^d1, Doxorubicin (50 mg/m2 in the first 4 cycles and 25 mg/m2 in the subsequent cycles) d1, Vincristine 1.4 mg/m^2 ^d1, and prednisone 50 mg/m^2 ^d1-5 (CHOP) regimen with good tolerance. He achieved complete clinical and radiological response and remained disease free 12 months after the end of chemotherapy treatment (Figure 5).

## Discussion

The maximum incidence of colorectal lymphomas is in the 50 to 70 year age group. The mean age at diagnosis was between 50 and 55 (3-83) years [3].

The standard criteria for the diagnosis of primary intestinal lymphomas were established by Dawson et al [4]. Tumours were considered to be primary on the following grounds: 1. The patient at first dose not present palpable superficial lymphadenopathy; 2. Chest radiographs showed no obvious enlargement of the mediastinal nodes; 3. The white blood cell counts, total and differential, were within normal limits; 4. At laparotomy the bowel lesion predominated, the only lymph nodes, obviously affected, are those in its immediate neighbourhood; 5. The liver and spleen appeared free of tumour in every case. Normal bone marrow biopsy examination and the absence of lymphadenopathy on computed tomographic (CT) scan of the mediastinun should be added to these criteria [3]. In the present case, the patient presented initially a heavily anorectal tumour followed by the involvement of the inguinal lymph nodes, which constitute the first lymphatic drainage of anal margin, without other radiological abnormalities. Tumour therefore fulfilled Dawson et al and Richards's criteria for primary colonic lymphoma.

The B cell lymphoma constitutes 85% of all primary colorectal lymphomas, with T cell lymphoma accounting for the remainder (15%). The differentiation between both lymphomas is done by the use of a cluser of differentiation antibodies (CD). The CD2, CD3, CD4, CD7 and CD8 are used to determine the T cell lymphoma. The CD20, CD79a, and CD10 are used to determine the B cell lymphoma [5]. The most common histological types of primary colorectal lymphomas, following the Revised European-American Classification of Lymphoid Neoplasms/World Health Organisation classification of lymphoid neoplasms (REAL/WHO), are: diffuse large B-cell lymphoma, Mantle-cell lymphoma and Burkitt's lymphoma [1]. In the case of our patient, most of the neoplasic cells were positive for CD-20 and for LCA antibody.

Because primary rectal lymphoma is rare, the optimal treatment for this disease remains to be more standardized. However, the role of surgery in the management of primary colorectal lymphoma is well defined. Surgical resection is the only statistically significant prognostic for patients with intestinal lymphomas [6]. It was reported that debulking surgery improves overall survival (OS) and disease free survival (DFS) [7]. In addition, 5-year survival rates were 46% for patients who underwent surgery vs 0% for patients managed without surgery [8]. The value of chemotherapy in case of intestinal lymphomas is less defined. In one series of colorectal lymphoma, all authors concluded that the surgery should be the primary treatment for localised disease; Chemotherapy and radiotherapy must be considered for advanced disease and for malignant lymphomatous poliposis [9]. Other authors showed a significant improvement in survival time (47.9 months vs 117.4 months) in patients with stage II high-grade colorectal lymphomas receiving adjuvant chemotherapy [10]. In another series, adjuvant chemotherapy after complete resection of colonic lymphoma improved significantly the disease free survival [11]. No series to date has reported experience with chemotherapy or radiotherapy as initial therapy for colonoscopically defined, surgically resectable primary colorectal lymphomas [12].

Although, the management of large bowel lymphoma involves a combination of surgery and chemotherapy, primary lymphoma of the rectum should be considered as a different clinico-pathological entity and treatment for this disease should be defined based on this special clinical condition. The value of surgery for primary lymphoma of the rectum is uncertain. In a review of 12 cases with lymphoma confined to the rectum (stage IE) reported by Mayo Clinic Centre, 5 patients undergone radical surgical resection and 7 patient received non surgical treatment. The authors showed that patients who had surgical excision did better than those treated nonoperatively [13]. Other authors confirmed the efficacy of surgery alone or in combination with radiotherapy or chemotherapy (Table 1). There have been rare cases managed with chemotherapy with or without radiotherapy (Table 1). Radiotherapy should be considered in mono therapy for the management of stage IE MALT (mucosa-associated lymphoid tissue) lymphoma and in combination with cytotoxic chemotherapy for the management of stage IE DLBCL (Table 1). Eradication of helicobacter pilory has also been used as an effective treatment of stage IE MALT lymphoma (Table 1).

**Table 1:** Clinical and pathological findings and outcome of selected published cases with stage IE primary NHL of the rectum managed with different strategies

Author	Year	Age (years)	Sex	Histological diagnosis	Stage	Treatment	Response	Follow-up	Status
Unal [5]	2008	43	Female	DLBCL	IE	CT → RT	CR	43	NED

Amouri [14]	2008	46	Female	MALT	IE	RT	CR	12	NED

Foo [15]	2008	60	Male	MALT	IE	RT	CR	41	NED

Guney [16]	2007	67	Female	DLBCL	IE	CT → RT	CR	12	NED

Wong [12]	2005	65	Male	Mantle	IE	S	CR	36	NED

Bilsel [17]	2004	33	Male	DLBCL	IE	CT → RT	CR	36	NED

Navarra [18]	2003	-	-	MALT	IE	S → CT	CR	36	NED

Matsumoto [19]	1997	-	-	MALT	IE	EHP	CR	3	NED

Shimono [20]	1995	58	Female	DLBCL	IE	RT+HT → S	CR	60	NED

For patients with widespread diseases the chemotherapy should be considered as logical treatment for colorectal lymphomas. The most widely chemotherapy used was CHOP (Cyclophosphamide 750 mg/m2 day 1, Doxorubicine 50 mg/m2 day 1, Vincristine 1.4 mg/m^2 ^day 1, and prednisone 50 mg/m^2 ^day 1 to 5) regimen [12]. Our patient with bulky disease was managed exclusively with CHOP chemotherapy with complete clinical and radiological response.

Using combined surgery and adjuvant chemotherapy, 5-year survival rates of patients with colorectal lymphoma have ranged from 27% to 55% [12].

## Conclusion

Lymphomas of the rectum should be considered as a different clinico-pathological entity. According to the literature, DLBCL of the large bowel must be treated with aggressive surgery followed by adjuvant chemotherapy. Although it is not possible to make a legitimate conclusion with a single case, we highlight the important role of chemotherapy for achieving successful treatment cure for patients with rectal DLBCL at stage IIEX. For this reason, the optimal treatment of this disease remains to be defined.

## Abbreviations

CT: chemotherapy; DLBCL: diffuse large B-cell lymphoma; EHP: eradication of helicobacter pylori; HT: hyperthermia; MALT: mucosa-associated lymphoid tissue; NED: no evidence of disease; RT: radiotherapy; S: surgery.

## Consent

Written informed consents were obtained from the patients for publication of this case series. A copy of the written consents are available for review by the journal's Editor-in-Chief.

## Competing interests

The authors declare that they have no competing interests.

## Authors' contributions

Alls authors have made significant contributions by making diagnosis and intellectual input in the case and writing the manuscript. all authors read and approved the final manuscript
